# Partial HIV C2V3 envelope sequence analysis reveals association of coreceptor tropism, envelope glycosylation and viral genotypic variability among Kenyan patients on HAART

**DOI:** 10.1186/s12985-017-0703-y

**Published:** 2017-02-14

**Authors:** Rose C. Kitawi, Carol W. Hunja, Rashid Aman, Bernhards R. Ogutu, Anne W. T. Muigai, Gilbert O. Kokwaro, Washingtone Ochieng

**Affiliations:** 1 Center for Research in Therapeutic Sciences, Strathmore University, P.O. Box 59857-00200, Nairobi, Kenya; 2 Jomo Kenyatta University of Agriculture and Technology, P.O Box 62000 -00200, Nairobi, Kenya; 3 South Eastern Kenya University, P.O Box 170-90200, Kitui, Kenya; 4 African Center for Clinical Trials, P.O. Box 2288-00202 Nairobi, Kenya; 5 Institute of Healthcare Management, Strathmore University, P.O. Box 59857-00200, Nairobi, Kenya; 6 Kenya Medical Research Institute, P.O. Box 54840-00200, Nairobi, Kenya; 7 Immunology and Infectious Diseases Dept, Harvard School of Public Health, Boston, MA USA

**Keywords:** HIV-1, Subtype, Potential N-linked glycosylation, Tropism, Treatment, Africa, Kenya

## Abstract

**Background:**

HIV-1 is highly variable genetically and at protein level, a property it uses to subvert antiviral immunity and treatment. The aim of this study was to assess if HIV subtype differences were associated with variations in glycosylation patterns and co-receptor tropism among HAART patients experiencing different virologic treatment outcomes.

**Methods:**

A total of 118 HIV env C2V3 sequence isolates generated previously from 59 Kenyan patients receiving highly active antiretroviral therapy (HAART) were examined for tropism and glycosylation patterns. For analysis of Potential N-linked glycosylation sites (PNGs), amino acid sequences generated by the NCBI’s Translate tool were applied to the HIVAlign and the N-glycosite tool within the Los Alamos Database. Viral tropism was assessed using Geno2Pheno (G2P), WebPSSM and Phenoseq platforms as well as using Raymond’s and Esbjörnsson’s rules. Chi square test was used to determine independent variables association and ANOVA applied on scale variables.

**Results:**

At respective False Positive Rate (FPR) cut-offs of 5% (*p =* 0.045), 10% (*p =* 0.016) and 20% (*p =* 0.005) for CXCR4 usage within the Geno2Pheno platform, HIV-1 subtype and viral tropism were significantly associated in a chi square test. Raymond’s rule (*p =* 0.024) and WebPSSM (*p =* 0.05), but not Phenoseq or Esbjörnsson showed significant associations between subtype and tropism. Relative to other platforms used, Raymond’s and Esbjörnsson’s rules showed higher proportions of X4 variants, while WebPSSM resulted in lower proportions of X4 variants across subtypes. The mean glycosylation density differed significantly between subtypes at positions, N277 (*p =* 0.034), N296 (*p =* 0.036), N302 (*p =* 0.034) and N366 (*p =* 0.004), with HIV-1D most heavily glycosylated of the subtypes. R5 isolates had fewer PNGs than X4 isolates, but these differences were not significant except at position N262 (*p =* 0.040). Cell-associated isolates from virologic treatment success subjects were more glycosylated than cell-free isolates from virologic treatment failures both for the NXT (*p =* 0.016), and for all the patterns (*p =* 0.011).

**Conclusion:**

These data reveal significant associations of HIV-1 subtype diversity, viral co-receptor tropism, viral suppression and envelope glycosylation. These associations have important implications for designing therapy and vaccines against HIV. Heavy glycosylation and preference for CXCR4 usage of HIV-1D may explain rapid disease progression in patients infected with these strains.

## Background

HIV-1 is highly genetically variable and on this basis, falls into four distinct groups: M, O, N and P [1, 2]. Group M viruses account for the majority of global HIV-1 infections and displays a tremendous amount of genetic variability as well, with nine divergent (pure) subtypes (A, B, C, D, F, G, H, J, and K), over 55 circulating recombinant forms (CRFs) and several unique recombinant forms (URFs) [3, 4]. These viruses also differ phenotypically in their co-receptor use preferences and entry to cells.

HIV-1 entry into host cells requires cooperate engagements of both the viral envelope and host cell surface (CD4) receptor, in a process requiring the engagement of one or more of a group of seven-transmembrane chemokine receptors (co-receptors), the CCR5 and CXCR4 being the most common [5, 6]. Viral tropism (co-receptor usage) has important consequences and relationships with infection and disease outcomes. Thus, viruses have been characterized phenotypically on the basis of tropism into syncytium inducing (SI) and non syncytium inducing (NSI) [7]. CXCR4 (X4) tropic viruses are largely of SI phenotype. These T-Cell tropic viruses arise during late stages of disease progression or infection [8]. CCR5 (R5) tropic viruses are of NSI phenotype of macrophage lineage and dominate the early stages of infection. HIV has demonstrated ability to switch tropism during the course of disease, a process that the virus uses adaptively to propagate in the presence of antiviral immune or therapeutic pressure [8]. Viral tropism can be determined either by using the more rigorous but expensive cell based phenotypic test or assigned on the basis of the relatively inexpensive genotyping sequence analysis that however, suffer from reduced sensitivity [9]. To improve robustness of genotypic tropism assignments, a number of tools have been developed including T-Cup 2.0, WebPSSM_x4r5_, WebPSSM_sinsi_ and Geno2Pheno, as well as rule-based methods like Esbjörnsson rule and Raymond’s rule with the objective of expanding the scope of sensitivity to include more non-B subtypes [9].

HIV is further adapted to its environment on the basis of envelope diversity, which exists in the form of variable glycosylation patterns and densities, as well as genetic variability affecting both immune and therapeutic functions [10]. The envelope consists of alternating constant regions (C1 - C5) and variable regions (V1 – V5) that provide critical structural and functional integrity for the virus, including co-receptor binding [11]. The overall amino acid charge of the HIV-1 V3 influences viral phenotype selection: a higher positive charge favors the SI phenotype and CXCR4 (X4) utilization while the loss of an N-linked glycosylation event in the V3 region together with a higher positive charge is associated with the virus switching from R5 to the X4 phenotype [6]. Overall, HIV-1 gp120 is heavily glycosylated by the infected host, with the glycans accounting for up to 50% of its total mass [12]. The position and number of N-Linked oligosaccharides attached to a protein have a profound effect on viral structure, protein expression and function [13], and specifically, tropism attributable to variations in the V3 loop [14]. These patterns also influence receptor binding and the phenotypic properties of the virus [6]. A change of sequons in the HIV envelope protein gp41, for example, can induce a conformational change in the associated gp120 that will dramatically diminish the binding of gp120-specific antibodies [15], and potentiating T cell immune escape [16, 17].

Studies have shown that genetic differences at HIV subtype level reflect in co-receptor usage and in the N-linked glycosylation pattern. Subtype C preferentially uses CCR5 and rarely induces syncytia [18]. Moreover, there is an apparent selection for subtype A and C variants that are less glycosylated and with shorter V1-V2 loop sequences [19], potentially enhancing the transmission of these strains. The faster rate of disease progression often accompanying HIV-1 subtype D can be linked to a higher frequency of syncytium formation and X4 usage [20].

Kenya’s HIV landscape features multiple genetic variants with at least 9 subtypes and recombinants [21–26], but only a few of local studies also looked at co-receptor and glycosylation characteristics across subtypes [27, 28]. In this paper, we sought to assess both phenotypic and genotypic differences in the context of expanded HAART so as to rapidly and readily generate comparative association matrix of potential N-linked glycosylation (PNG) pattern, co-receptor usage and viral subtype diversity in relation to clusters of virologic treatment outcome. Partial *C2V3* envelope sequences generated previously from our published work was used for these analyses [29].

## Methods

### Subject Characteristics

Associations and patterns of co-receptor usage relative to virus subtype and treatment outcomes were assessed from sequences and data derived from 59 patients receiving HAART from various Kenyan clinical settings as described in our previous publication [29]. Due to resource limitation, and in order to align objectives with primary treatment outcomes, sequences were generated only for viral load (VL) clusters of patients with VL greater than 1000 copies and those with VL = < 1000 to correspond to virologic treatment failure (VF) and virologic treatment success (VS) respectively, with at least 6 months interval of two successive VL (VL1 and VL2) testing. These virologic treatment outcome clusters were described in our earlier paper [29]. The sequences of those with VS were obtained from PBMC (proviral DNA) while those of VF were obtained from plasma (viral RNA). This clustering strategy further allowed us to assess sequence diversity by blood compartment and by virologic treatment response. Demographic data, ART history and VL1 were obtained from clinic medical records and by using a structured questionnaire as applicable. Treatment protocol and ART regimens at the time of the study [30], followed national guidelines that have recently been revised [31]. ART regimens included various combinations of zidovudine (AZT), lamivudine (3TC), stavudine (D4T), nevirapine (NVP), efavirenz (EFV), tenofovir (TDF) and Abacavir (ABC), with majority of the patients initiating first line D4T + 3TC + NVP (ca.48%), followed 26% on the AZT + 3TC + NVP regimen. The rest (26%) initiated either TDF + 3TC + NVP (24%) or ABC + 3TC + NVP (2%) regimen combinations. About 54% of all the patients had already switched regimen from the initiation regimen to a new substitution first-line regimen. These cases were associated largely with poor clinic record-tracking and patient medication disclosure or recall.

### Subtype sequence analysis for coreceptor usage

We have previously reported viral nucleotide sequence information based on *C2V3* region of HIV-1 obtained from 59 patients enrolled from Comprehensive Care Centers across Kenya [29]. These sequences were subtyped using JPHMM - Jumping Profile Hidden Markov Model - (http://jphmm.gobics.de) and on the basis of phylogenetic analysis. The two methods of subtyping were found to be comparable for subtype assignment except for recombinant viruses for which phylogenetic method is least built to detect. In this paper, all subtypes are reported on the basis of JPHMM assignment to include recombinant strains. These sequences, which are available at the GenBank under the accession numbers: KM853037 - KM853095, are used in this publication to determine envelope glycosylation and tropism. Overall, input sequence identity ranged from 82% on the lower side to 95% on the higher side relative to reference sequence.

### Determination of viral tropism

For optimal determination of tropism of different HIV subtypes, the use of multiple tools becomes more relevant due to sensitivity limitation of any single genotyping tool, particularly when dealing with non-B HIV subtypes [9, 32, 33]. Therefore, we applied multiple genotyping tools to infer co-receptor usage of the virus isolates that were subtyped on the basis of both JPHMM and phylogeny. For comparison of co-receptor usage and HIV subtype in this paper, JPHMM-assigned subtypes were used as basis, as this method also was able to determine recombinant subtypes and has a better diagnostic odds ratio for subtype A that predominates Kenyan population [9]. Aligned and edited sequences that covered the entire V3 region (*n* = 57/59) were input to the geno2pheno platform [34] (http://coreceptor.geno2pheno.org/index.php) under the section that allows for examination of co-receptor usage. Similarly, the sequences were applied to WebPSSM [35], Raymond rule [33], Esbjörnsson rule [32] and Phenoseq [36]. For Geno2Pheno, we first applied a false positive rate (FPR) cut-off of 10% (FPR10), being the standard recommendation of the ‘European Consensus Group on clinical management of HIV-1 tropism testing’. Since we only generated a single sequence for each virus isolate, we repeated this analysis with FPR set at 20% (FPR20) to increase fidelity for correctly assigning X4-phenotype, and at 5% FPR (FPR5) to accommodate emerging subtype C variants in Kenya, given recent findings from subtype C HIV-1 phenotyping showing an increased confidence at identifying CXCR4-usage in both CXCR4-exclusive and dual tropic variants at this FPR cut-off. [37] In all cases, values below set cut-off were considered to be predictive of an X4 virus. Differences in the outputs of co-receptor usage (inferred phenotype) were finally compared for association with assigned subtype using chi square tests.

### Determination of Potential N-Glycosylation sites (PNGs)

We next picked 55 of the 57 sequences spanning the entire C2V3; these 55 were of comparable nucleotide length to allow unbiased quantitative and qualitative PNG determinations. Analysis of PNGs was done by inputting the selected envelope sequences into the Los Alamos HIV platform, and applying specific database tools to generate different PNG matrices (http://www.hiv.lanl.gov/content/sequence/HIV/HIVTools.html). Briefly, the nucleic acid sequences were first translated into amino acid sequences using the TRANSLATE tool. The generated amino acid sequences were then aligned against HXB2 reference sequence using HIVAlign (the genomic region chosen was *env*). The aligned sequences were then applied to the N-GLYCOSITE tool to generate an array of PNG patterns (http://www.hiv.lanl.gov/content/sequence/GLYCOSITE/glycosite.html). We used the HXB2 reference strain for numbering of glycosite positions and the NXT, NXS and contiguous NNXT (S) patterns to develop a PNG report matrix. Data for all the analyses are presented either as graphical or tabular outputs, with numerical mean values representing number of PNG sites identified. PNGs patterns were compared across different viral strains for different amino acid positions and used to deduce the extent of envelope protein divergence between subtype isolates.

### Statistical data analyses

Data was grouped into categorical and scale variables that included number of PNGs and VL (scale variables), and coreceptor tropism, PNG pattern, and nucleic acid (NA) source material for the isolates (categorical variables). Because patients were sampled purposively on the basis of VL (hence virologic treatment failure or virologic treatment success groups), the use of VL as scale variable was limited to when comparisons were made within subgroups but not between groups of categorical variables. For viral tropism, the subjects were grouped into 2 categories as R5-tropic or X4-tropic (no R5X4 dual tropic strains were observed). ANOVA was used to assess the difference in the distribution of scale variable between groups of independent variables. Specifically, ANOVA was used to compare the number of PNGs across subtype, phenotype (viral tropism) and NA source. For this part, PNG variables were presented as either (i) the total PNGs, (ii) the specific numbers of each NXT, NXS and NNXS (T) PNG patterns and (iii) in terms of amino acid positions that had a high proportion of sequences having a glycosylation site at that position (greater than 60%). Association between two independent variables were examined using a Chi Square test. For this part, analyses strategy focused on comparing the proportion of variable events within and across groups. P-values are reported at the 0.05 significance level. Data that were not significant are mostly not shown even if mentioned.

## Results

### Associations of viral subtype diversity and coreceptor tropism

The data presented here build on our previous study and includes virus sequences that are already published for HIV subtype analyses [29]. Two of the 59 sequence isolates did not span the entire *V3* region and were thus deemed too short for coreceptor analysis. Four of the 59 (including 2 with short V3 region), had C2V3 region too short for phylogenetic subtyping, but were still able to be subtyped based on JPHMM, REGA, geno2pheno (G2P), and NCBI bioinformatics platforms. JPHMM based subtypes were selected for further comparative data analysis, because of its larger concurrence with phylogeny for assigning pure subtypes, and for its superior detection of recombinants. Of the 57 sequences with complete V3 region and using JPHMM,36 (~63.1%) were pure subtype A1, 7 (~12.3%) were recombinants of A1, 4 (7%) were A2, 5 (8.8%) each were subtype C and subtype D. Three different FPR cut-offs (5%, 10% and 20%) were applied on the G2P platform for sequence-based coreceptor usage analysis as described under methods. Forty-three of the 57 sequences (75%) were CCR5 (R5) tropic and 14 (25%) were CXCR4 (X4) tropic viruses at FPR of 10% (FPR10). No dual tropic (R5X4) virus isolates were found using the Geno2Pheno phenotyping algorithms. For all three FPR cut-off algorithms, there was a significant relationship between viral subtype and viral tropism (χ^2^ *p =* 0.045; *p =* 0.016 and *p =* 0.005 for FPR5, FPR10 and FPR20 respectively). Clustered by viral subtype, approximately 80% of subtype A1 sequences were R5 tropic, against 20% X4 subtype A1 viruses. Similarly, a larger proportion (ca. 75%) of A1 recombinant viruses were R5 tropic. Unlike subtype A1 viruses, there were only four A2 viruses, split equally betweenX4 and R5 phenotype. None of the subtype C viruses were of X4 phenotype using all three FPR algorithms, compared to nearly all (80% at FPR10 and 100% at FPR20) of subtype D viruses being found to be X4-tropic. These analyses suggested a trend whereby more of the pure subtype A1 were likely to be assigned as R5 than as X4 tropic at lower FPR, while more of the pure subtype D isolates tended to be assigned as X4 tropic at higher FPR. Tropism of pure subtype C and A2 viruses appeared unaffected across FPR algorithms, while that of recombinant strains either varied minimally or not at all. Table 1 shows the distribution of viral tropism across HIV subtype variants at the 3 FPR cut-offs.

**Table 1:** The distribution by number and (proportions), of viral tropism across HIV subtype variants using Geno2Pheno at three cut-off points

Viral Tropism at FPR cut-off							
	FPR, 5%		FPR (10%)		FPR (20%)		TOTAL
HIV-1 Subtype	CCR5,	CXCR4,	CCR5,	CXCR4,	CCR5,	CXCR4, N	
A1	31 (86.1%)	5 (13.9%)	29 (80.6)	7 (19.4)	26 (72.2)	10 (27.8)	36 (100)
^a^A1r.	6 (77.8)	1 (22.2)	6 (85.7)	1 (14.3)	6 (85.7)	1 (14.3)	7 (100)
A2	2 (50)	2 (50)	2 (50)	2 (50)	2 (50)	2 (50)	4 (100)
C	5 (100)	0 (0)	5 (100)	0 (0)	5 (100)	0 (0)	5 (100)
D	2 (40)	3 (60)	1 (20)	4 (80)	0 (0)	5 (100)	5 (100)
χ^2^ * p* value	*0.045*		*0.016*		*0.005*		
Total	46 (81)	11 (19)	43 (75)	14 (25)	39 (68)	18 (32)	57 (100)

^a^A1r, recombinants of subtype A1. The A1 recombinants included A1D (3), A1A2 (2),A1H (1), A1A2D (1). The number of sequence isolates for each category of co-receptor tropism is shown followed by the percentages in parentheses. *P*-value is derived from cross-tabulation of co-receptor tropism and virus subtype

We also applied alternate algorithms including (Raymond’s rule [33], Esbjörnsson’s rule [32], WebPSSM [35] and Phenoseq [36]) to assess the distribution and examine the association between subtype and tropism (Table 2). These analyses revealed significant associations for Raymond’s rule (*χ* ^2^; *p =* 0.024), for WebPSSM_x4r5_ (*p =* 0.05) but not for Phenoseq and Esbjörnsson’s rule. The largest deviation in coreceptor usage assignment was related to subtype A. Overall, Esbjörnsson and Raymond’s rules scored more subtype A1 strains as X4 tropic than the rest of the platforms while WebPSSM scored most strains as R5 tropic. Phenoseq assigned all (100%) of the A2 subtypes as R5 tropic, which were only 50% R5 by other platforms. Esbjörnsson also assigned one subtype C and at least 10% of A1 viruses as X4 tropic, which were otherwise R5 tropic by all other methods used. G2P concurred with Raymond’s rule on the tropism assignment for subtype D. We did not conduct direct phenotype testing to validate against sequence-based phenotype derivations.

**Table 2:** Associations of co-receptor tropism by various methods of assignment, with HIV subtype

		Subtype: Number of isolates, (proportion (%)					Total	*χ* ^2^; *p*-value
		A1	A1R	A2	C	D		
WebPSSM	CCR5	32 (88.9)	7 (100)	2 (50)	5 (100)	3 (60)	49 (86)	0.05
	CXCR4	4 (11.1)	0 (0)	2 (50)	0 (0)	2 (40)	8 (14)	
Total		36 (100)	7 (100)	4 (100)	5 (100)	5 (100)	57 (100)	
Raymond	CCR5	28 (77.8)	6 (85.7)	2 (50)	5 (100)	1 (20)	42 (73.7)	0.024
	CXCR4	8 (22.2)	1 (14.3)	2 (50)	0 (0)	4 (80)	15 (26.3)	
Total		36 (100)	7 (100)	4 (100)	5 (100)	5 (100)	57 (100)	
Esbjörnsson	CCR5	25 (69.4)	6 (85.7)	2 (50)	4 (80)	2 (40)	39 (68.4)	0.435
	CXCR4	11 (30.6)	1 (14.3)	2 (50)	1 (20)	3 (60)	18 (21.6)	
Total		36 (100)	7 (100)	4 (100)	5 (100)	5 (100)	57 (100)	
Phenoseq	CCR5	30 (83.3)	6 (85.7)	4 (100)	5 (100)	3 (60)	48 (84.2)	0.416
	CXCR4	6 (16.7)	1 (14.3)	0 (100)	`	2 (40]	9 (15.8)	
Total		36 (100)	7 (100)	4 (100)	5 (100)	5 (100)	57 (100)	
G2PFPR10	CCR5	29 (80.6)	6 (85.7)	2 (50)	5 (100)	1 (20)	43 (75.4)	0.016
	CXCR4	7 (19.4)	1 (14.3)	2 (50)	0 (0)	4 (80)	14 (24.6)	
Total		36 (100)	7 (100)	4 (100)	5 (100)	5 (100)	57 (100)	

### Distribution of potential N-linked glycosylation sites (PNGs) at specific amino acid positions

In analyzing the pattern and distribution of PNGs, only those sequences whose subtype could be determined phylogenetically were considered because these sequences were of approximately equal length hence bias due to differences in sequence length would be minimized. Fifty-five of the 59 sequences could be defined phylogenetically [29] and were therefore analyzed for PNGs patterns as described under methods. In terms of specific amino acids (A.A), the largest numbers of virus isolates were glycosylated at asparagine (N) position 277 (98.2%) and at N302 (98.2%) (Table 2). This was true whether the distribution was assessed within viral tropism clusters (R5 and X4 viruses), or within the different nucleic acid source material. Specifically, the proportion of sequences with PNGs at specific A.A. positions was 80% (44/55) for N262 and 98.2% (54/55) for N277. The rest were: N296 (38/55, 69.1%), N302 (54/55, 98.2%), N337 (33/55, 60%), N345 (40/55, 72.7%), N366 (41/55, 74.5%), N399 (43/55, 78.2%) and N408 (40/55, 72.7%). Assessed by viral tropism, N277, N302 and N366 were the most dominant PNG sites for R5 tropic isolate, compared to N262, N277 and N302 for X4 viruses. These patterns remained largely true when data was further disaggregated by nucleic acid source as either being derived from DNA or RNA material as presented in Table 3.

**Table 3:** The distribution and clustering of specific amino acid PNG sites according to viral tropism and source material

Number of isolates; % possessing the shown amino acid PNG at specified site										
	N262	N277	N296	N302	N337	N345	N366	N399	N408	Total
NA source										
*DNA*	33; 82.5	40; 100	28; 70	39;97.5	25;62.5	30; 75	32; 80	35;87.5	33; 82.5	40; 100
*RNA*	11; 73.3	14;93.3	10;66.7	15; 100	8; 53.3	10;66.7	9; 60	8; 53.3	7; 46.7	15; 100
Tropism										
*CCR5*	31; 73.8	41;97.6	29;69.0	41;97.6	25;59.5	30;71.4	33;78.6	31;73.8	29; 69.0	42; 100
*CXCR4*	13; 100	13;100	9; 69.2	13;100	8; 61.5	10;76.9	8; 61.5	12;92.3	11; 84.6	13; 100
Subtype										
A1	28; 77.8	36; 100	24; 66.7	36; 100	18; 50	27; 0.75	30; 83.3	25; 69.4	26; 72.2	36; 100
A1r^a^	4; 80	4; 80	5; 100	5; 100	4; 80	3; 60	2; 40	4; 80	3; 60	5; 100
A2	3;75	4;100	4;100	4;100	3;75	2;50	3;75	4;100	2;50	4;100
C	4;80	5;100	1;20	4;80	4;80	3;60	5;100	5;100	4;80	5;100
D	5;100	5;100	4;80	5;100	4;80	5;100	1;20	5;100	5;100	5;100
Overall	44; 80	*54;98.2*	*38;69.1*	*54;98.2*	33; 60	40;72.7	*41;74.5*	43;78.2	40; 72.7	55;100

Key: *NA* Nucleic Acid. Tropism analysis is based on false positive rate of 10%. ^a^A1r; A1A2 (*n* = 1), A1D (*n* = 3), and A1A2D (*n* = 1). ﻿﻿Mean PNG differences are significant between subtypes at specific A.A positions N277 (*p* = 0.034), N296 (*p* = 0.036), N302 (*p* = 0.034) and N366 (*p* = 0.004) in Anova tests

Glycosylation positions N277 and N302 occurred in all subtype A1 isolates, representing 100% proportional glycosylation at these amino acid position. By similar analogy, 100% glycosylation of isolates was attributed to positions N296 and N302 for A1 recombinants; N277, N297, N302 and N399 for subtype A2; N277, N366 and N399 for subtype C and at N262, N277, N302, N345, N399 and N408 for subtype D. By comparison, only 20% for subtype D isolates against 100% for subtype C isolates were glycosylated at N366. These results reveal amino acid preference for glycosylation of different HIV subtypes, and are presented in Table 3. Subtype D was most heavily glycosylated, with PNGs occurring at 8 out of the 9 amino acid positions examined. An ANOVA test to compare mean PNGs for the different subtypes at specific amino acid position yielded significant results for positions N277 (*p =* 0.034), N296 (*p =* 0.036), N302 (*p =* 0.034) and N366 (*p =* 0.004).

### The pattern of potential N-linked glycosylation sites (PNGs) according to subtype, tropism and nucleic acid source material

For this analysis, viral genetic diversity was categorized into the five observed subtypes as A1, recombinants of A1, A2, C and D. The PNG patterns were NXT, NXS and NNX (S) T. Table 4 shows the details for the average number of pattern-specific PNGs disaggregated by viral subtype, tropism and source material. For the 55 sequences with complete C2V3 sequence region, the total number of PNGs considering all the three possible patterns was 615 (mean, 11.18, standard error –SE -, 0.274). Of the total PNGs, a majority (65.5%, or 403/615) were of NXT type (mean, 7.33, SE 0.239) while 33.3% (205/615) were of NXS type (mean, 3.73, SE 0.167). Only a smaller proportion of PNGs (1.14%) were of the contiguous NNXS (T) pattern (mean, 0.13, SE 0.052). With the exception of NNXS (T) PNG that was rare, each of the subtype isolates was glycosylated at the very least, at a position corresponding to each of NXT and NXS PNG patterns (denoted by 100% in table 4). When all the possible PNG patterns were further considered by virus subtype, the mean PNGs for A1 was 11.11 (SE 0.367), A1 recombinants was 10.80 (SE 0.970), A2 was 10.75 (SE 0.629), C was 11.20 (SE 0.663) and D was 12.40 (SE 0.748). Thus subtype D isolates had the largest number of PNGs per isolate while recombinant strains had the least. Next, we considered each of the individual PNG patterns instead of just the total, and compared their mean distribution across virus subtype. Similarly, subtype D was the most abundantly glycosylated at NXT sites (mean of 8.4; SE 0.400), with A1 strains being least glycosylated at these sites. Glycosylation density was comparable at NXS sites for subtypes A1 (mean 3.78; SE 0.204), A1 recombinants (mean 3.80,SE 0.663) subtypes C (mean, 3.80; SE 0.583) and subtypes D (mean, 3.80; SE 0.374) but much less for subtype A2 (mean, 3.00; SE 0.913). As expected, glycosylation at the contiguous NNXS (T) sites was the least common and was observed at a mean occurrence of 0.14 (SE, 0.071) per isolate for A1 viruses, 0.20 (SE 0.20) for A1 recombinants, and 0.2 (SE 0.2) for D viruses. None of the subtype C or A2 isolates were glycosylated at the NNXS (T) site. When analysed by viral tropism, X4-tropic isolates were more glycosylated than the R5-tropic isolates except for the NNX (S) T pattern. This difference though, was not significant for individual patterns.

**Table 4:** Average PNGs per isolate for each subtype, nucleic acid source and viral tropism

	Mean PNGs, Number of isolates; % of *n*			
Number of isolates, (% of Total)	All Patterns	NXT Pattern	NXS Pattern	NNX (S) T Pattern
Subtype				
Subtype A1, *n* = 36 (65.4)	11.11, 36; 100	7.19, 36; 100	3.78, 36; 100	0.14, 4; 11.1
A1r recombinants *n* = 5 (9.1)	10.80, 5; 100	6.80, 5; 100	3.80, 5; 100	0.20, 1; 20
Subtype A2, *n* = 4 (7.3)	10.75, 4; 100	7.75, 4; 100	3, 4; 100	0, 0; 0
Subtype C, *n* = 5 (9.1)	11.2, 5; 100	7.4, 5; 100	3.8, 5; 100	0, 0; 0
Subtype D, *n* = 5 (9.1)	*12.4, 5; 100*	*8.4, 5; 100*	*3.8, 5; 100*	*0.2, 1; 20*
Tropism				
CCR5, *n* = 42 (76.4)	11.02, 42; 100	7.21, 42; 100	3.64, 42; 100	0.17, 5; 100
CXCR4, *n* = 13 (23.6)	11.69, 13; 100	7.69, 13; 100	4, 13; 100	0
Nucleic Acid Source				
*DNA, n = 40 (72.7)*	*11.6* ^a^, *40; 100*	*7.68* ^a^, *40; 100*	3.85, 40; 100	0.08, 2; 5
*RNA, n = 15 (27.3)*	*10.07* ^a^, *15; 100*	*6.4* ^a^, *15; 100*	3.4, 15; 100	0.27, 3; 20
Total, *n* = 55 (100)	11.18, 55; 100	7.33, 55; 100	3.73, 55; 100	0.13, 5; 9.1

Key: A1R: recombinants of A1 (A1A2, *n* = 1; A1D, *n* = 3;& A1A2D, *n* = 1); ^a^Significantly different between DNA (cellular) and (RNA) extracellular viral isolates for the NXT (*p* = 0.016), and for all the patterns﻿ combined (*p* = 0.011)﻿﻿

### The relative envelope glycosylation pattern and density in cellular and extracellular matrixes

**Fig. 1:**
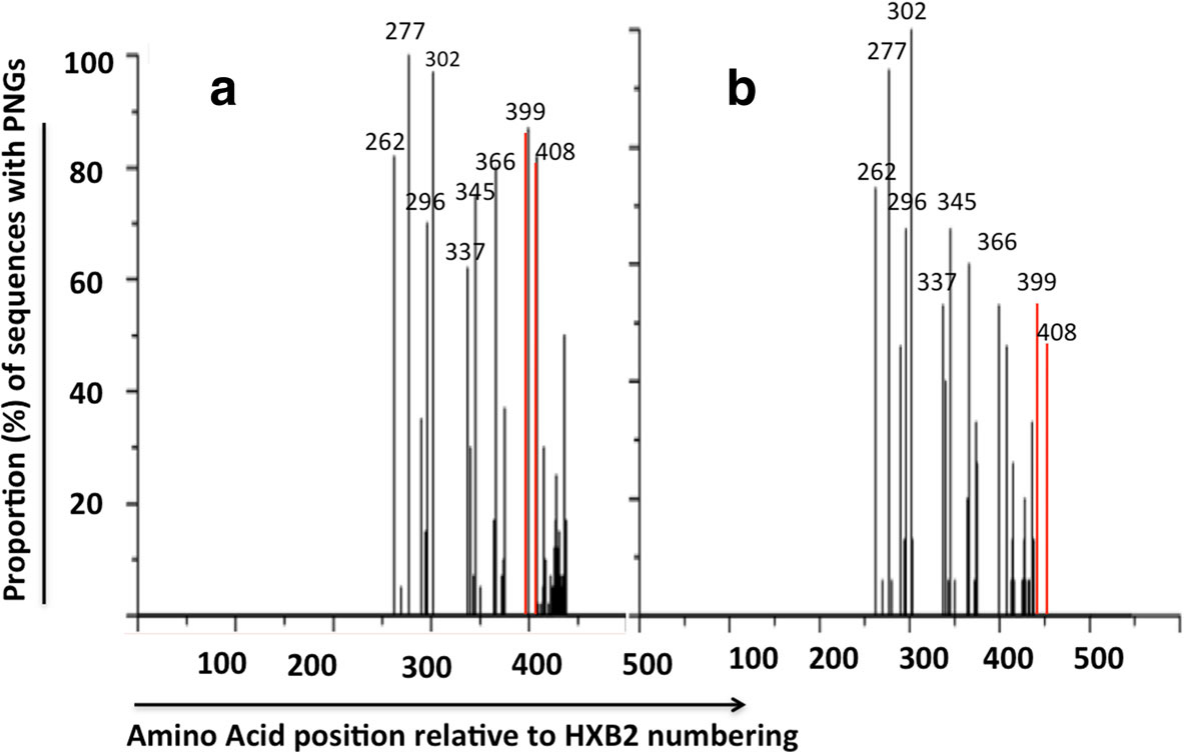
Proportion of isolates with PNGs at specific amino acid position relative to HXB2 reference sequence. Data is shown for isolates derived from cellular blood compartment (**a**), and those derived from cell-free blood (plasma) (**b**). Each vertical spike represents the proportion of sequences glycosylated at that specific sequence position. The cell-associated virus isolates are proportionally more glycosylated (more longer vertical spikes) than cell-free isolates. Red spikes show amino acid position (N399 and N408) at which glycosylation differed significantly between extracellular and cell-derived isolates

Nucleic Acid (NA) source material was used as a proxy for viral compartmentalization in blood, with plasma-derived RNA isolates being representative of extracellular blood compartment and PBMC-derived DNA isolates representing cellular compartment. It was also used as a proxy for virologic response, as all plasma derived isolates were drawn from virologic treatment failures and cellular isolates from responders. Clustered by blood compartment, there were on average, 12 PNGs for each viral isolate derived from DNA (cellular) compartment compared to 10 PNGs from each isolate of RNA (extracellular) material. DNA derived isolates were still the most glycosylated at NXT sites. Analysis of variance (ANOVA) conducted to compare mean values of the specific PNG patterns across NA source revealed a significantly different pattern and density of PNG between cellular and extracellular viral isolates for the NXT glycosylation pattern (*p =* 0.016), and for all the patterns combined (*p =* 0.011). Moreover considering specific amino acid PNG sites, there was still a significant difference in glycosylation between cellular and extracellular isolates at A.A positions N399 (*p =* 0.006) and N408 (*p =* 0.007). These data are presented partially in Fig. 1.

## Discussion

The HIV virus establishes lifelong infection despite the use of HAART, and in many instances, ‘escape’ therapeutic intervention because of continuous viral evolution and formation of quasi species with distinct genetic and phenotypic variability. In these patients on extensive HAART in a context of highly dynamic and heterogeneous virus populations, different viral subtypes and recombinants exhibit diversity in coreceptor tropism as well as glycosylation patterns. We discuss these results that have important implication to HIV pathogenesis and therapeutic targeting [38–41].

### Viral tropism and co-receptor usage

HIV-1 tropism is critical to host-cell interactions and is implicated widely in disease or infection processes. Our data revealed a significant association between viral subtype and coreceptor tropism using G2P platform (all FPR algorithms), Raymond’s rule and WEBPSMM. Between 68% and 81% of all the isolates were R5-tropic using G2P’s three FPR algorithms. None of the isolates were determined to be dual tropic across all criteria used. FPR10 is routinely applied as the standard algorithm for HIV-1 tropism determination by G2P, but is biased in favor of subtype B and likely to over-represent R5-tropism in settings where HIV is highly genetically variable like Kenya. Therefore, we applied different phenotyping approaches to assess tropism of the different isolates. There was still however, substantial discordance among the alternative phenotyping algorithms in assigning tropisms to the different subtypes. Specifically, output from Raymond’s and Esbjörnsson’s platforms tended to have higher proportions of X4 variants, while output from WebPSSM had lower proportions of X4 variants relative to other algorithms used. Actual phenotyping tests were not done to validate the accuracy of these phenotyping platforms, thus limiting the breadth of interpreting the efficiencies of the different platforms for correct phenotype assignment.

Evidence suggests that different HIV subtypes may have specific preferences for coreceptor usage [42]. Subtype C for example has been shown to preferentially use CCR5 and to rarely induce syncytia [43, 44]. A few studies involving Kenyan subjects have shown majority of circulating HIV to be R5 tropic, and mostly due to the predominance of R5-tropic HIV subtype A [27, 28, 45]. Significant variations became apparent when tropism is disaggregated by viral subtype, with majority of subtype A1, C and A1 recombinants being R5 tropic. The converse was true for subtype D viruses, nearly all of which were X4-tropic. CXCR4 usage is preferred by subtype D viruses and is generally associated with a more rapid decrease of CD4 counts and rapid disease progression [20, 27]. Subtype D, C and recombinant HIV strains have shown specific dynamism in Kenya over the past decade, partly due to population and demographic cross-border patterns, with the ultimate effect on a shrinking predominance of subtype A viruses [21, 29, 46, 47]. In this setting of highly variable viral genetic strains and where adherence and virologic treatment failure is prevalent [48], the outcomes of long-term treatment is likely to be additionally influenced by the changing phenotypic property that promotes rapid disease processes. In particular, a rise in X4-tropic subtype D would imply increased risk of rapid disease and potential dampening of antiretroviral treatment (ART) response. Thus, treatment plans will need to be structured to account for both viral genetic diversity and the associated phenotypic characteristics.

Apart from subtype C, there has not been much success in coming up with highly specific and sensitive phenotyping or genotyping tools for the other non-B subtypes. For subtype A for example, one study showed that for the multiple tools used, specificity was high but sensitivity was very low [9]. The low sensitivity was likely attributable to other regions outside the V3 (i.e. V1, V2 and V4) which also impact tropism [9, 49, 50]. Thus accurate assignment of tropism would require an expanded analysis of the envelope, beyond the V3 as considered in this paper. Such accuracy becomes significant in the design and management of therapy, given certain CCR5 blockers have failed in patients co-infected with X4-tropic viruses [51].

### Potential N-Linked Glycosylation Sites (PNGs)

The HIV-1 is heavily coated with glycans that make the virus ‘invisible’ to the host’s immune system [52]. An increase in the length and number of PNGs in the V1V2 region for example has been shown to play a role in HIV-1 resistance to neutralizing antibodies [53]. On average, we found that, the NXT PNG pattern was significantly compartmentalized in blood, with heavy NXT glycosylation resident in the cellular compartment of patients that responded effectively to HAART. This preferential glycosylation for the NXT relative to NXS pattern has been documented by others as well [54]. Contrary to our observations, independent data from the analysis of C2-V5 gp120 envelope has shown more glycosylation of RNA isolates from plasma than of proviral DNA from cellular compartment in Australian patients on HAART [55]. Our data was derived from a much shorter V3 region and in addition, varies from the Australian finding in that all our isolates from cellular blood were obtained from patients who had suppressed virus to undetectable levels hence experiencing long-term virologic treatment success (VS), and all the cell-free isolates were from patients with virologic treatment failure (VF). We hypothesized that long-term exposure to HAART that results in VS would dim the need for robust humoral response against the virus that is already under intense antiretroviral pressure. The result is a reduced selection pressure for antibody escape, and the propagation of viruses that are more likely to lose their PNGs than those from VF patients where robust antibody response is needed to raise alternative antiviral defense. This line of argument is supported by earlier observations that HIV-specific antibody responses are depressed in both chronically infected and in HAART responsive patients [56]. Similarly but unlike the previous study, our population was infected with multiple virus subtypes with the main strain being HIV subtype A1, which is unlike HIV infection environment in most western countries where subtype B predominates with little to no genetic heterogeneity of virus populations [57].

N-linked glycosylation constitute both structural and functional adaption of the virus that affect a range of host infection outcomes including transmissibility and immune evasion [58, 59]. Another likely but more cautious argument is that accumulation of HIV isolates with fewer PNGs in cell free plasma could suggests a ‘molecular learning’ and protective process by which HIV-1 sheds off specific glycosites under therapeutic pressure. Ultimately, such an association of reduced PNG sites in plasma virus isolates retain credibility when considered in the context of accompanying antibody neutralization [59]. When we further disaggregated isolates by viral subtype and by tropism, HIV-1D and X4-tropic isolates tended to have higher average PNGs per isolate than other subtypes or R5-tropic strains. Since both subtype D and X4 tropism are associated with adverse disease outcomes, increased glycosylation of these strains may be associated with specific protective amino acid sites that reinforce the mechanisms for HIV pathogenesis. In deed our data supports this argument, as there was absolute (100%) glycosylation of X4 tropic viruses at N262 relative to other strains. This position was recently reported to provide steric hindrance in antibody neutralization experiments that protected the virus from antibody targeting [60]. Subtype D also had the highest number of PNG sites glycosylating 100% of the isolates compared to other subtypes. Some evidence from mainly subtype B and C viruses have suggested that R5-tropic strains tend to have more PNGs than their X4 counterparts [61, 62]. There is certainly a need to interpret these data within the various specific contexts of HIV infection. In populations with highly heterogeneous genetic diversity of HIV, infections with multiple strains and variable treatment approaches and outcomes, it is not unlikely for the virus to acquire different adaptation strategies.

Glycosylation at position 301 (302) at the stem of the V3 loop is thought to play a role in stabilizing the V3 loop and in modulating co-receptor binding. Mutation at this point results in a drastic decrease in viral infectivity probably due to reduced co-receptor binding [12]. In addition to positions N296 and N366, our analyses revealed significant differences by subtype at glycosylation position N302. At position 296, only 20% of the subtype C isolates were glycosylated compared to 64.7% for subtype A1, 80% for subtype D and 100% for subtype A2 or of recombinants of A1. Conversely, 100% of subtype C sequences were glycosylated at N366 compared to only 20% of subtype D, 57% of A1 recombinants, 75% of subtype A2 and 82% of subtype A1. At position N302, all the subtypes had 100% glycosylation except for subtype C that had 80% glycosylation. Hence, envelope molecular patterns that affect HIV glycosylation varies across viral genotypes, and may have association with disease or treatment outcome. Overall, our data should be interpreted cautiously within the context limitations of the study; the sample size was not large enough to warrant population-level inferences. Similarly due to limitation in available resources, we did not sequence DNA of patients experiencing virologic treatment failure (VL > 1000) nor generate RNA derived sequences of those with virologic treatment success (VL < =1000). Including these sequences in future comparisons may add important dimensions as regards viral diversity between proviral reservoirs and circulating clones. This study however, provides useful basis for deeper investigations of larger representative samples from long-term HAART patients.

## Conclusions

Kenya and most countries where HIV treatment was for a long time limited by the availability of resources to fund basic antiretroviral medication, has in the last 10-12 years scaled up treatment without equivalent programs to monitor important outcomes and effects of the same. We and a few others have recently dedicated research to evaluate these effects, in respect of adherence, treatment outcomes and genetic diversity of the HIV in the presence of HAART [29, 48, 63]. The present set of data demonstrates that in these patients who have received suppressive HAART over an extended period, viral tropism and glycosylation follows a trajectory of viral genetic diversity, compartmentalization and virologic response. Specifically, genetic variability at subtype level was significantly associated with virus preference for co-receptor usage and with envelope glycosylation pattern and density- subtype D being preferentially X4-tropic, extracellularly compartmented in blood and more heavily glycosylated than other subtypes. Furthermore, envelope glycosylation is significantly associated with virus tropism, compartmentalization in blood and virologic treatment response. As discussed elsewhere in this paper, these relationships are not just consequential to treatment options, but also to therapeutic and preventive vaccine designs.

## Abbreviations

ANOVA: Analysis of variance
CREATES: Centre for research in therapeutic sciences
CRF: Circulating recombinant forms
DNA: Deoxyribonucleic Acid
Env: HIV Envelope
ERC: Ethical review committee
FPR: False positive rate
HAART: Highly active anti-retroviral therapy
HIV-1: Human immunodeficiency virus type 1
JPHMM: Jumping profile hidden markov model
KEMRI: Kenya medical research institute
NA: Nucleic acid
NSI: Non syncytium inducing
PBMC: Peripheral blood mononuclear cells
PNG (s): Potential N-linked Glycosylation (sites)
RNA: Ribonucleic acid
RT-PCR: Reverse transcriptase polymerase chain reaction
SI: Syncytium inducing
SSC: Scientific steering committee
URF: Unique recombinant forms
VF: Virological treatment failure
VL: Viral load
VS: Virological treatment success
